# Structure and Solvent Effects on Nonconventional Fluorescence in Choline–Carboxylic Acid Ionic Liquids Hybrid Solvents

**DOI:** 10.1021/acs.jpcb.6c05016

**Published:** 2026-09-09

**Authors:** Solomon Owiredu, Aleah Berkebile, Grace Carr, Tashinga Chigodora, Juan Martinez Soto, Nicole McGuire, Duran Lee, Samuel A. Oluwole, J. S. Raaj Vellore Winfred, Christian Agatemor

**Affiliations:** † Department of Chemistry, 4517 Bucknell University, Lewisburg, Pennsylvania 17837, United States; ‡ Department of Chemistry and Biochemistry, 7823 Florida State University, Tallahassee, Florida 32306, United States

## Abstract

The molecular parameters that influence emissive-state formation and excited-state relaxation in nonconventional fluorophores remain poorly understood. Here, we use structurally related choline–carboxylic acid ionic liquids (ILs) derived from valeric acid (CAVA), levulinic acid (CALE), and 4-pentenoic acid (CAPE) to investigate how the structure, composition, and solvent environment of the carboxylic acid influence nonconventional fluorescence. CAVA exhibits negligible fluorescence, whereas CALE and CAPE display pronounced and composition-dependent emission in aqueous solution. Increasing the choline:carboxylic acid ratio from 1:1 to 1:4 enhances the observed fluorescence intensity in both emissive series, while replacing the carbonyl-containing levulinic acid with alkenyl-containing 4-pentenoic acid markedly alters the emission profile, excitation dependence, and excited-state dynamics. SAXS reveals no detectable nanoscale aggregation associated with fluorescence enhancement, while viscosity and ^1^H NMR measurements show composition-dependent changes in bulk and local intermolecular environments. Neither properties nor differences in excitationlight absorption consistently track the fluorescence behavior across CALE and CAPE. Solvent- and excitation-dependent fluorescence, ultrafast transient absorption, and time-resolved fluorescence measurements reveal excitation-dependent emission, sub-picosecond spectral evolution, and biexponential nanosecond decay, consistent with a complex excited-state landscape. Together, these results demonstrate that carboxylic acid structure, composition, and solvent environment substantially influence the photophysical behavior of these ILs and establish choline–carboxylic acid ILs as a useful platform for investigating structure–photophysics relationships in nonconventional fluorescence.

## Graphical abstract

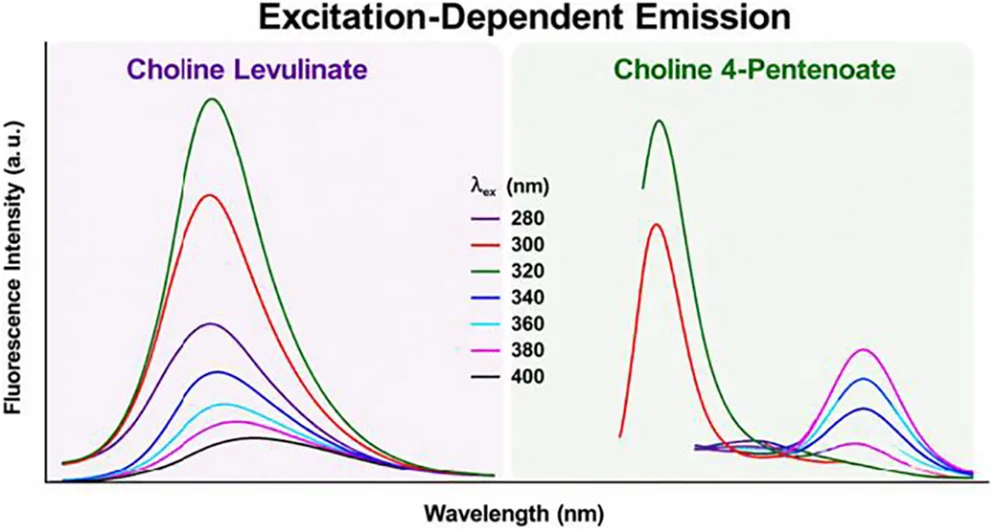

## 1 Introduction

The discovery of nonconventional fluorescence in nonconjugated and nonaromatic molecules fundamentally expands the structural space accessible to fluorophores and challenges conventional paradigms of molecular emission.^1–10^ These findings motivate ongoing efforts to elucidate how weak electronic interactions arising from local clustering and intermolecular organization give rise to emissive excited states in nonconventional fluorophores.^7,8^ Over the past decade, accumulating experimental and theoretical evidence has revealed key photophysical characteristics that distinguish conventional from nonconventional fluorophores. For example, excitation-dependent emission, multiple emissive states, mismatched absorption and excitation spectra, and pronounced sensitivity to the local microenvironment now define many nonconventional fluorescent systems.^7,8^ Further, mechanistic investigations of nonconventional fluorophores increasingly implicate through-space electronic interactions, clusterization-triggered emission, and conformational rigidification in emissive-state formation.^7,8^ Despite these advances, the field still lacks a predictive molecular framework linking molecular structure and local organization to emissive-state formation, excited-state relaxation, and fluorescence behavior.^11,12^ Consequently, the challenge now extends beyond discovering new nonconventional fluorophores to establishing molecular-level design principles for rationally tuning their photophysical properties. Although aggregation and clusterization remain central to current mechanistic models, growing evidence indicates that relatively subtle structural modifications can also reshape emissive-state landscapes.^13^ Yet, most molecular engineering strategies have focused on introducing additional heteroatoms, donor–acceptor motifs, or conformational constraints.^13^ By comparison, comparatively little is known about how functional group substitution within an otherwise conserved molecular framework influences nonconventional fluorescence. Systematic studies that isolate these molecular-level effects, therefore, remain scarce, leaving unresolved the question of how functional group and local intermolecular interactions influence emissive-state formation and excited-state relaxation independently of aggregation.

A related and equally important unresolved question concerns how the local microenvironment influences nonconventional fluorescence.^13^ In conventional fluorophores, extensive experimental and theoretical studies have established clear relationships between solvent properties, excited-state electronic structure, and emission behavior.^14^ By contrast, although numerous studies demonstrate that the solvent environment strongly influences nonconventional fluorescence,^15–17^ the mechanistic understanding of these effects remains considerably less well understood than in conventional fluorophores. Consequently, understanding how molecular structure and the local microenvironment jointly shape emissive-state formation, excited-state relaxation, and fluorescence behavior remains a central challenge in the development of predictive structure–photophysics relationships for nonconventional fluorophores.

To this end, we employed choline–carboxylic acid ionic liquids (ILs) as a modular model platform to investigate nonconventional fluorescence. This platform permits independent modification of its constituent components within a common molecular framework,^18,19^ enabling systematic evaluation of how molecular structure and local microenvironment influence photophysical behavior. This platform permits systematic variation of molecular structure and composition, which can alter hydrogen-bonding interactions, ion pairing, and local electronic environments relevant to emissive-state formation and excited-state relaxation.^20–22^ Within this platform, we selected valeric acid, levulinic acid, and 4-pentenoic acid because they constitute a well-defined series of C5 carboxylic acids that enables systematic evaluation of how introducing carbonyl and alkenyl functionalities into an otherwise conserved molecular framework influences nonconventional fluorescence. Using steady-state and time-resolved spectroscopic techniques, we demonstrate that functional groups within the carboxylic acid structure and solvent environment strongly influence fluorescence intensity, excitation dependence, and excited-state dynamics. Collectively, these findings establish choline–carboxylic acid ILs as a versatile platform for elucidating structure–photophysics relationships in nonconventional fluorophores. They further demonstrate that variations in carboxylic acid structure can substantially alter the photophysical behavior within this molecular series.

## 2 Materials and Methods

### 2.1 Materials

Choline bicarbonate solution (80 wt % in H_2_O, Sigma-Aldrich), valeric acid (≥99%, Sigma-Aldrich), levulinic acid (99%, Sigma-Aldrich), and 4-pentenoic acid (97%, Sigma-Aldrich) were used as received without further purification. LC–MS-grade methanol and acetonitrile, as well as molecular biology-grade dimethyl sulfoxide (DMSO), were purchased from Sigma-Aldrich and used for solvent-dependent spectroscopic studies. Deionized (DI) water (18.2 MΩ·cm) was used for all aqueous sample preparations. Deuterated dimethyl sulfoxide (DMSO-*d*_6_) was purchased from Cambridge Isotope Laboratories and used as the solvent for NMR measurements.

### 2.2 Preparation and Characterization of ILs

The ILs were synthesized using a modified version of a previously reported method.^23–25^ Briefly, the appropriate carboxylic acid (valeric acid, levulinic acid, or 4-pentenoic acid) was combined with choline bicarbonate at the desired molar ratio and stirred at room temperature until CO_2_ evolution ceased, indicating completion of the acid–base neutralization reaction (Figure 1). Water was removed by rotary evaporation at 60 °C for 30 min, and the resulting products were further dried in a vacuum oven at 60 °C for 48 h. The resulting ILs were stored in a desiccator until use. The water content of the IL was not quantified. Although all samples were subjected to the same drying and storage conditions to maintain consistent sample preparation, possible effects of residual water in the IL on intermolecular organization and fluorescence cannot be excluded. The identity of the ILs was confirmed by ^1^H and ^13^C NMR spectroscopy (Supporting Information). The ^1^H and ^13^C NMR spectra were recorded on a Varian 400 MHz NMR spectrometer. Samples were prepared by dissolving the ILs in DMSO-*d*_6_ to a final concentration of 400 mM.

**Figure 1:**
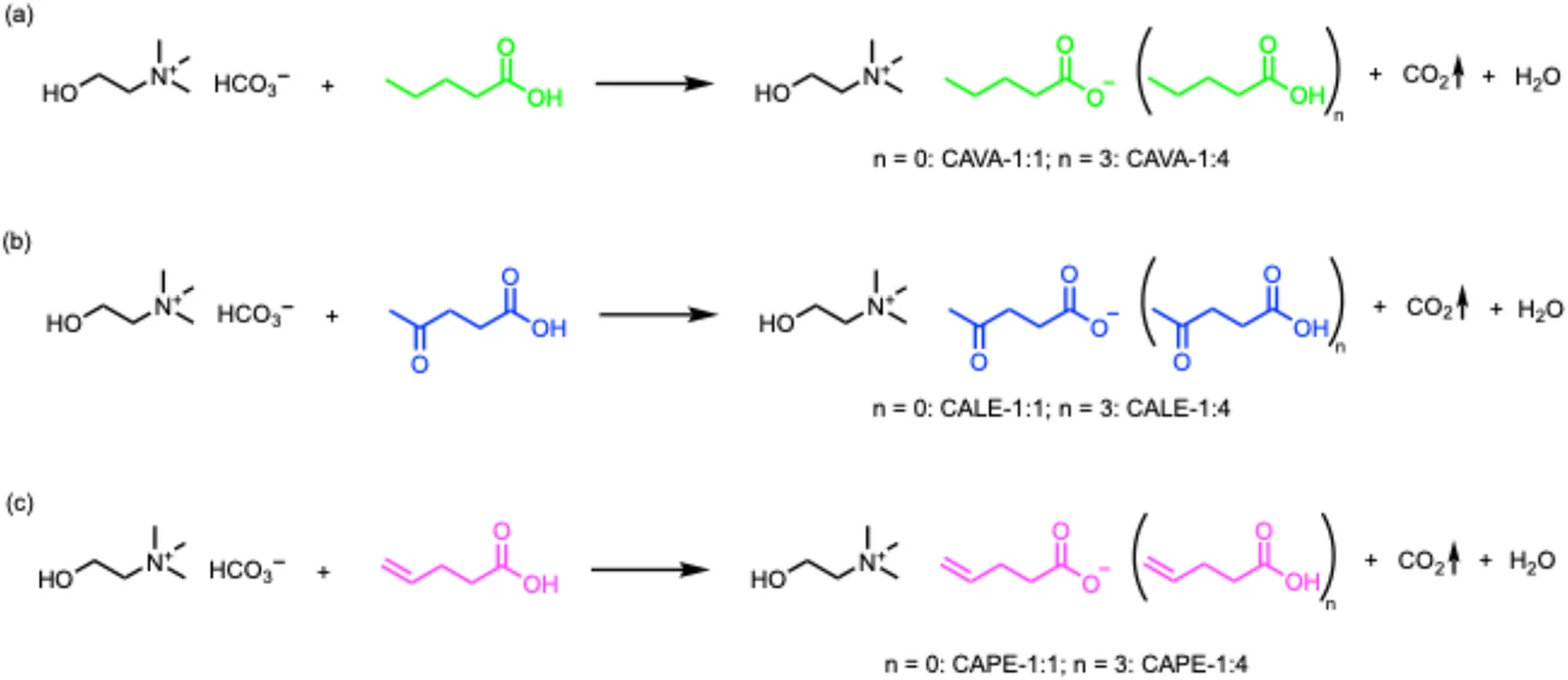
Synthesis of the choline–carboxylic acid ionic liquids. Choline bicarbonate was reacted with (a) valeric acid, (b) levulinic acid, or (c) 4-pentenoic acid to produce the corresponding ionic liquids CAVA, CALE, and CAPE through acid–base neutralization. The choline-to-carboxylic acid molar ratio (*n*) was adjusted to prepare the 1:1 (*n* = 0) and 1:4 (*n* = 3) compositions. The three C5 carboxylic acids provide a systematic molecular series that varies the functionality within a common carbon framework, enabling evaluation of how saturated, carbonyl-containing, and alkenyl-containing structures influence nonconventional fluorescence.

### 2.3 UV–Visible Absorption Spectroscopy

UV–visible absorption spectra were recorded using an Agilent Cary UV–Vis Compact spectrophotometer equipped with a 10 mm × 10 mm quartz cuvette (Hellma Analytics), with DI water used as the reference blank. Spectra were collected over the wavelength range of 240–700 nm and processed using OriginPro (OriginLab Corp.).

### 2.4 Steady-State Fluorescence Spectroscopy

Fluorescence emission and excitation spectra were acquired using a Cary Eclipse fluorescence spectrophotometer equipped with a quartz microcell (Type 115F-QS, Hellma Analytics; high-performance quartz glass, pathlength 10 mm × 2 mm, sample volume 400 μL, PTFE stopper, wavelength range 200–2500 nm). The excitation and emission slit widths were set to 5 nm, spectra were collected at a scan rate of 600 nm min^–1^, with an averaging time of 0.1 s and a data interval of 1 nm. Excitation-dependent emission spectra were collected by varying the excitation wavelength while monitoring the resulting fluorescence emission. Excitation spectra were obtained by monitoring fluorescence at selected emission wavelengths. Sample concentrations and solvent compositions are specified in the corresponding figure captions. Spectral data were processed and plotted using OriginPro (OriginLab Corp.).

### 2.5 Photoluminescence Quantum-Yield Measurements

Absolute photoluminescence quantum yields were measured at the Florida State University Department of Chemistry and Biochemistry Spectroscopy Laboratory using a Hamamatsu Quantaurus-QY spectrometer. The instrument was equipped with a 150 W xenon arc lamp excitation source and an integrating sphere for absolute quantum-yield determination. Samples were excited at 320 nm, and the resulting emission spectra were collected over the visible spectral region. Quantum yields were calculated from the ratio of emitted to absorbed photons using the instrument’s integrated-sphere method and associated Quantaurus analysis software. DI water was measured under identical conditions and used as a blank reference for background correction. Absorbance and emission spectra were recorded simultaneously, and photoluminescence quantum yields were obtained directly from the instrument software.

### 2.6 Time-Resolved Fluorescence Spectroscopy

Fluorescence lifetime measurements were performed at the Florida State University Department of Chemistry and Biochemistry Spectroscopy Lab using an Edinburgh Instruments LP980 spectrometer operating in the time-correlated single-photon counting (TCSPC) mode. Decay profiles were recorded at the samples’ emission maxima after excitation at 320 nm and analyzed by iterative reconvolution with the measured instrument response function. Fluorescence decays were fitted using a biexponential decay model, and amplitude-weighted average lifetimes were calculated from the fitted lifetime components and amplitudes. Spectral and kinetic data were analyzed using Edinburgh Instrument’s LP980 software and OriginPro (OriginLab Corp.). The LP980 system was equipped with a Hamamatsu R928 photomultiplier tube detector (200–870 nm) and operated with a time calibration of 0.200 ns channel^–1^.

### 2.7 Femtosecond Transient Absorption Spectroscopy

Ultrafast transient absorption measurements were performed at the Florida State University Department of Chemistry and Biochemistry Spectroscopy Lab using a HELIOS femtosecond transient absorption spectrometer. The system was driven by a Ti regenerative amplifier (Coherent Astrella, 100 fs pulse duration, 1 kHz repetition rate) coupled to an optical parametric amplifier (Coherent OPerA Solo) providing pump wavelengths from 290 to 2600 nm. Samples were excited at 320 nm and probed over the wavelength range of 340–600 nm using a 2 mm quartz cuvette. The probe beam consisted of a white-light continuum detected with a 1024-pixel CMOS array detector (spectral response 200–1000 nm). The pump power was maintained at 1.2 mW, spectra were averaged for 2.0 s per acquisition, and measurements were acquired with an 8 ns time window. The optical delay line provided a temporal resolution of approximately 150 fs with a delay-line resolution of 14 fs. Raw transient absorption data were processed using Surface Xplorer software (Ultrafast Systems), including background subtraction, chirp correction, time-zero correction, principal component reconstruction, baseline correction, and conversion to differential absorbance. Processed data were subsequently analyzed and plotted using OriginPro (OriginLab Corp.) to evaluate excited-state relaxation dynamics.

### 2.8 Small-Angle X-ray Scattering (SAXS)

SAXS measurements were performed on aqueous IL solutions (400 mM) and DI water as a solvent blank using a Xenocs Xeuss 2.0 system at the Dual-Source and Environmental X-ray Scattering (DEXS) facility at the University of Pennsylvania. Samples were loaded into sealed quartz capillaries and measured in transmission geometry at room temperature under identical experimental conditions. Data were collected using a GeniX3D Cu Kα X-ray source (8 keV, λ = 1.54 Å) and a Pilatus 1M detector. Each sample, including the blank, was measured with an exposure time of 1800 s. Two-dimensional scattering patterns were azimuthally integrated using XSACT Pro software to generate one-dimensional scattering intensity profiles as a function of the scattering vector, q. The scattering vector was defined as *q* = (4π sin θ)/λ, where θ is one-half of the scattering angle (2θ), and λ is the X-ray wavelength. Solvent blank measurements were collected under identical conditions and used for comparison.

### 2.9 Viscosity Measurements

The viscosities of 400 mM aqueous IL solutions were determined at 21.7 ± 0.2 °C using a RheoSense microVISC viscometer. Measurements were conducted under steady laminar-flow conditions in accordance with the manufacturer’s recommended operating procedures. Instrument calibration was verified using a NIST-traceable viscosity standard (Cannon N7.5), which was analyzed before and after sample measurements to confirm measurement accuracy and instrument stability. Individual samples were introduced into the device using disposable microVISC pipettes (RheoSense) to minimize contamination and ensure reproducible sample loading. Each solution was measured independently three times, and the reported viscosity values represent the average of these measurements, with the corresponding standard deviation.

## 3 Results and Discussion

### 3.1 Variations in Carboxylic Acid Structure Reshape the Emission Behavior in ILs

An unresolved challenge in the emerging field of nonconventional fluorescence is understanding how molecular structure and the local microenvironment govern emissive-state formation and excited-state relaxation. To examine the contribution of molecular structure, we investigated how carboxylic acid structure influences nonconventional fluorescence using the well-defined choline–carboxylic acid IL series CAVA, CALE, and CAPE (Figure 1a–c). In these systems, the 1:1 compositions represent the choline carboxylate ILs, whereas the 1:4 compositions are acid-rich IL hybrid systems containing excess carboxylic acid. In a related choline carboxylate IL system, we previously demonstrated that increasing the carboxylic acid content reorganizes intermolecular interactions, including hydrogen bonding, while preserving ionic character.^26^ This precedent suggests that carboxylic acid enrichment in the present 1:4 compositions may similarly alter the intermolecular interactions relative to the corresponding 1:1 compositions. Therefore, such changes may be relevant to through-space interactions implicated in nonconventional fluorescence,^7,8^ although their contribution to the observed emission is not established by the present study.

The fluorescence response depends strongly on the identity of the carboxylic acid structure. At an identical concentration (400 mM), both CAVA-1:1 and CAVA-1:4 (Figure 1a) display negligible fluorescence (Figure 2a,b). In contrast, CALE exhibits pronounced fluorescence centered at approximately 426 nm (λ_ex_ = 300 nm), with increasing levulinic acid content to form CALE-1:4 (Figure 1b), producing an approximately twofold increase in the observed emission intensity (Figure 2a,b). At 300 nm, the corresponding 400 mM solutions exhibit absorbances of 1.55 and 3.86 for CALE-1:1 and CALE-1:4, respectively (Figure SI1). These high optical densities limit the quantitative interpretation of relative fluorescence intensities due to potential inner-filter effects. Likewise, CAPE displays a more pronounced compositional response, with increasing 4-pentenoic acid content to form CAPE-1:4 (Figure 1c), producing an approximately 14-fold increase in observed fluorescence intensity relative to CAPE-1:1 (Figure 2a,b). The corresponding absorbance at 300 nm increases from 0.32 to 1.15 (Figure SI1), indicating that increased excitationlight absorption may contribute to the observed change in fluorescence intensity. However, the increase in absorbed excitation light is substantially smaller than the observed fluorescence response, indicating that differences in absorption alone do not explain the pronounced composition-dependent emission of CAPE. Overall, the absorption and emission responses of both CALE and CAPE vary substantially with composition.

**Figure 2:**
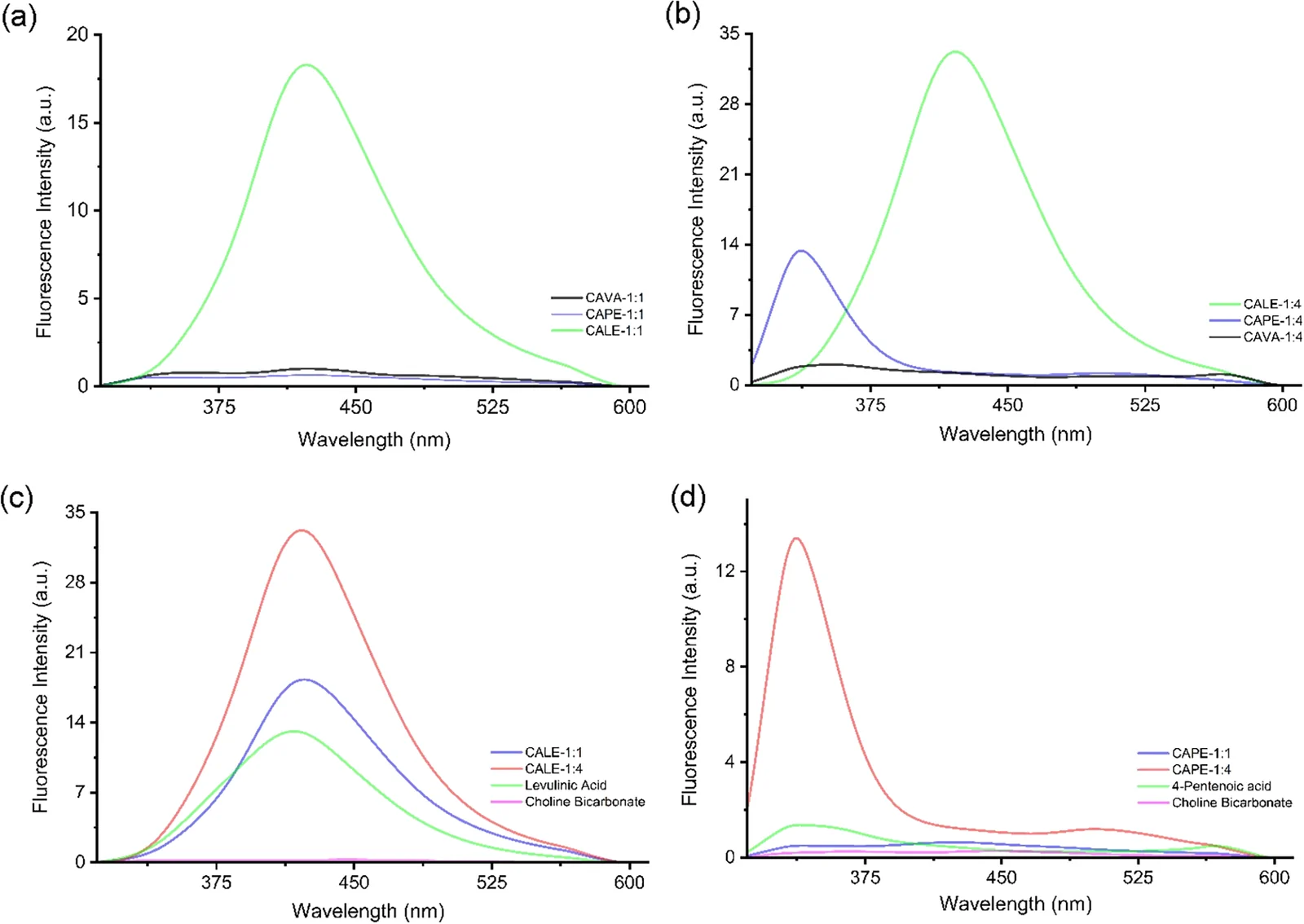
Influence of carboxylic acid structure and ionic-liquid composition on the fluorescence behavior of choline–carboxylic acid ionic liquids. (a,b) Emission spectra (λ_ex_ = 300 nm) of 400 mM aqueous solutions of the CAVA, CALE, and CAPE ionic liquids at choline:carboxylic acid molar ratios of 1:1 (a) and 1:4 (b). (c,d) Emission spectra (λ_ex_ = 300 nm) comparing 400 mM aqueous solutions of CALE (c) and CAPE (d) with their corresponding molecular precursors, levulinic acid and 4-pentenoic acid, respectively, and choline bicarbonate. At λ_ex_ = 300 nm, the absorbances of the 400 mM solutions were 1.55 (CALE-1:1), 3.86 (CALE-1:4), 0.32 (CAPE-1:1), and 1.15 (CAPE-1:4).

Further, CAPE-1:4 exhibits a markedly different emission profile from that of CALE-1:4. Whereas CALE-1:4 displays a single broad emission band centered near 426 nm, CAPE-1:4 exhibits a higher-energy emission maximum near 360 nm together with a broad lower-energy shoulder extending to approximately 520 nm (Figure 2a,b). Because valeric acid, levulinic acid, and 4-pentenoic acid share comparable C5 carbon frameworks and the same carboxylic acid head group, the contrasting emissive behaviors are, therefore, consistent with an influence of the functionality embedded in the carboxylic acid framework. Valeric acid contains only a saturated alkyl chain; levulinic acid and 4-pentenoic acid incorporate carbonyl and alkenyl functionalities, respectively. The electronic features of the functional group may alter through-space interactions, including *n*···*n* and *n*···π interactions implicated in nonconventional fluorescence,^7,8^ although the specific interactions underlying the observed emission remain unresolved. Collectively, these findings demonstrate that variations in carboxylic acid structure substantially influence nonconventional fluorescence in choline–carboxylic acid ILs. They also provide a new perspective on our previous observations in choline–geranic acid ILs, in which the coexistence of carbonyl and alkenyl functionalities precluded assessment of their individual contributions to the observed nonconventional fluorescence.^23^ More broadly, the findings extend emerging evidence that anion identity and intermolecular interactions influence the photophysical behavior of choline-based ILs and deep eutectic solvents.^27,28^

The pronounced differences among CAVA, CALE, and CAPE indicate that carboxylic acid structure is an important molecular variable influencing the nonconventional fluorescence. Accordingly, we next asked whether these differences simply reflect the intrinsic fluorescence of the constituent molecules or arise from changes in their photophysical behavior upon IL formation. Because CAVA exhibited negligible fluorescence under the conditions examined (Figure 2a,b), subsequent mechanistic investigations focused on the emissive CALE and CAPE systems. Comparison of CALE and CAPE with their molecular precursors reveals that IL formation substantially reshapes the fluorescence behavior of the precursors (Figure 2c,d). CALE-1:1 exhibits substantially stronger fluorescence than either levulinic acid or choline bicarbonate, together with a red-shifted emission maximum relative to both precursors, indicating that IL formation modifies both the intensity and spectral characteristics of the emission (Figure 2c). In contrast, although 4-pentenoic acid is weakly fluorescent, CAPE-1:1 exhibits even lower emission intensity, whereas increasing the 4-pentenoic acid content to form CAPE-1:4 produces a pronounced enhancement in fluorescence (Figure 2d). Thus, the fluorescence behavior of the ILs cannot be predicted solely from the intrinsic emission of their molecular precursors; rather, it emerges from the interplay between molecular structure and IL composition. We next examined whether differences in excitationlight absorption contribute to the distinct fluorescence responses of CALE and CAPE by comparing their solvent-blank-corrected UV–visible absorption spectra (Figure SI1). At 300 nm, the 400 mM solutions exhibit absorbances of 1.55 and 3.86 for CALE-1:1 and CALE-1:4, respectively, and 0.32 and 1.15 for CAPE-1:1 and CAPE-1:4, respectively, measured using a 10 mm path-length cuvette. The relatively high absorbances, particularly for the CALE samples, increase susceptibility to inner-filter and reabsorption effects; consequently, a shorter 2 mm path-length cuvette was used for all fluorescence measurements to reduce these optical effects. The differences in absorbance at 300 nm indicate that excitationlight absorption may contribute to the observed fluorescence intensities. However, comparison of the absorption and emission data shows that absorbance at the excitation wavelength alone is insufficient to account for the distinct fluorescence responses. Notably, CALE-1:1 and CAPE-1:4 have comparable absorbances at 300 nm (1.55 and 1.15, respectively) yet exhibit markedly different fluorescence intensities and emission profiles (Figure 2a,b). CALE-1:1 emits predominantly above 375 nm, whereas CAPE-1:4 exhibits an emission maximum below 375 nm. These results indicate that molecular structure and composition affect both light absorption and the photophysical response of these ILs following excitation.

Replacing the carbonyl-containing levulinate in CALE with the alkenyl-containing 4-pentenoate in CAPE is expected to alter the local electronic environment, hydrogen-bonding interactions, solvation, ion pairing, and bulk liquid properties, all of which can influence emissive-state formation and excited-state relaxation.^21,22,29,30^ Among these factors, viscosity is frequently associated with enhanced nonconventional fluorescence because increased microenvironmental rigidity restricts molecular motions and suppresses nonradiative decay.^31–33^ To assess its potential contribution, we measured the viscosities of the aqueous IL solutions. CALE-1:1, CALE-1:4, CAPE-1:1, and CAPE-1:4 exhibited viscosities of 1.090 ± 0.009, 1.465 ± 0.031, 1.156 ± 0.017, and 1.730 ± 0.011 mPa·s, respectively. Within both IL series, increasing the carboxylic acid content increased both viscosity and fluorescence intensity (Figures 2a,b). Specifically, the approximately twofold increase in fluorescence observed for CALE-1:4 relative to CALE-1:1 was accompanied by a 34% increase in viscosity, whereas the approximately 14-fold enhancement observed for CAPE-1:4 was accompanied by a 50% increase in viscosity. These trends establish an association between viscosity and fluorescence intensity within each series, consistent with a possible contribution from increased microenvironmental rigidity. A caveat, however, is that viscosity alone does not explain the pronounced photophysical differences between the two IL families. Despite exhibiting a slightly higher viscosity than CALE-1:1, CAPE-1:1 displays substantially weaker fluorescence. Likewise, the fluorescence enhancement observed upon transitioning from CAPE-1:1 to CAPE-1:4 is not commensurate with the corresponding increase in viscosity relative to the CALE series. These observations indicate that the positive association between viscosity and fluorescence intensity observed within a given series does not extend across the two IL systems. Thus, although increased viscosity accompanies the fluorescence enhancement within each series, the relationship between viscosity and fluorescence differs between CALE and CAPE, further linking the observed photophysical behavior to carboxylic acid structure.

Clusterization is a widely invoked mechanism for nonconventional fluorescence, whereby molecular association restricts conformational motion, promotes through-space electronic interactions (e.g., *n*···*n* and *n*···π interactions), and suppresses nonradiative relaxation, thereby facilitating emission.^8,34^ Consistent with this framework, numerous molecular and polymeric systems exhibit a correlation between nonconventional fluorescence and nanoscale aggregation.^2,9,35–38^ Likewise, previous studies of choline-based ILs, including our work on choline–geranic acid systems, identified nanoscale organization.^23,25,39–42^ We therefore examined whether the fluorescence observed in the CALE and CAPE series similarly correlates with nanoscale self-assembly. Small-angle X-ray scattering (SAXS) measurements were performed on 400 mM aqueous solutions of CALE-1:1, CALE-1:4, and CAPE-1:4, and the results were compared with those of deionized water under identical conditions. After transmission correction using the measured transmitted-beam intensity relative to the empty-beam intensity, none of the IL solutions exhibited a pronounced low-*q* upturn, correlation peak, or other scattering feature indicative of nanoscale aggregation beyond that of water (Figure 3a). Therefore, the substantial fluorescence enhancement observed for the 1:4 compositions is not accompanied by SAXS-detectable nanoscale ordering. Although SAXS cannot exclude transient molecular associations, short-range organization below its detection limit, or dynamically fluctuating intermolecular contacts, the data argue against a mechanism requiring large, persistent aggregates or phase-separated clusters as a prerequisite for fluorescence enhancement. At first glance, the absence of SAXS-detectable aggregation appears to contrast with our previous observations in choline–geranic acid ILs,^23^ where TEM revealed pronounced nanoscale organization. However, this difference most likely reflects the distinct self-assembly propensities of the constituent carboxylic acids rather than fundamentally different photophysical mechanisms. Geranic acid possesses a substantially more hydrophobic and amphiphilic framework than either levulinic acid or 4-pentenoic acid and therefore exhibits a greater tendency to form nanoscale assemblies in aqueous media. These findings establish that nanoscale self-assembly in choline–carboxylic acid ILs is highly structure-dependent. More importantly, they demonstrate that SAXS- or TEM-detectable aggregation is not a universal requirement for nonconventional fluorescence or its enhancement.

**Figure 3:**
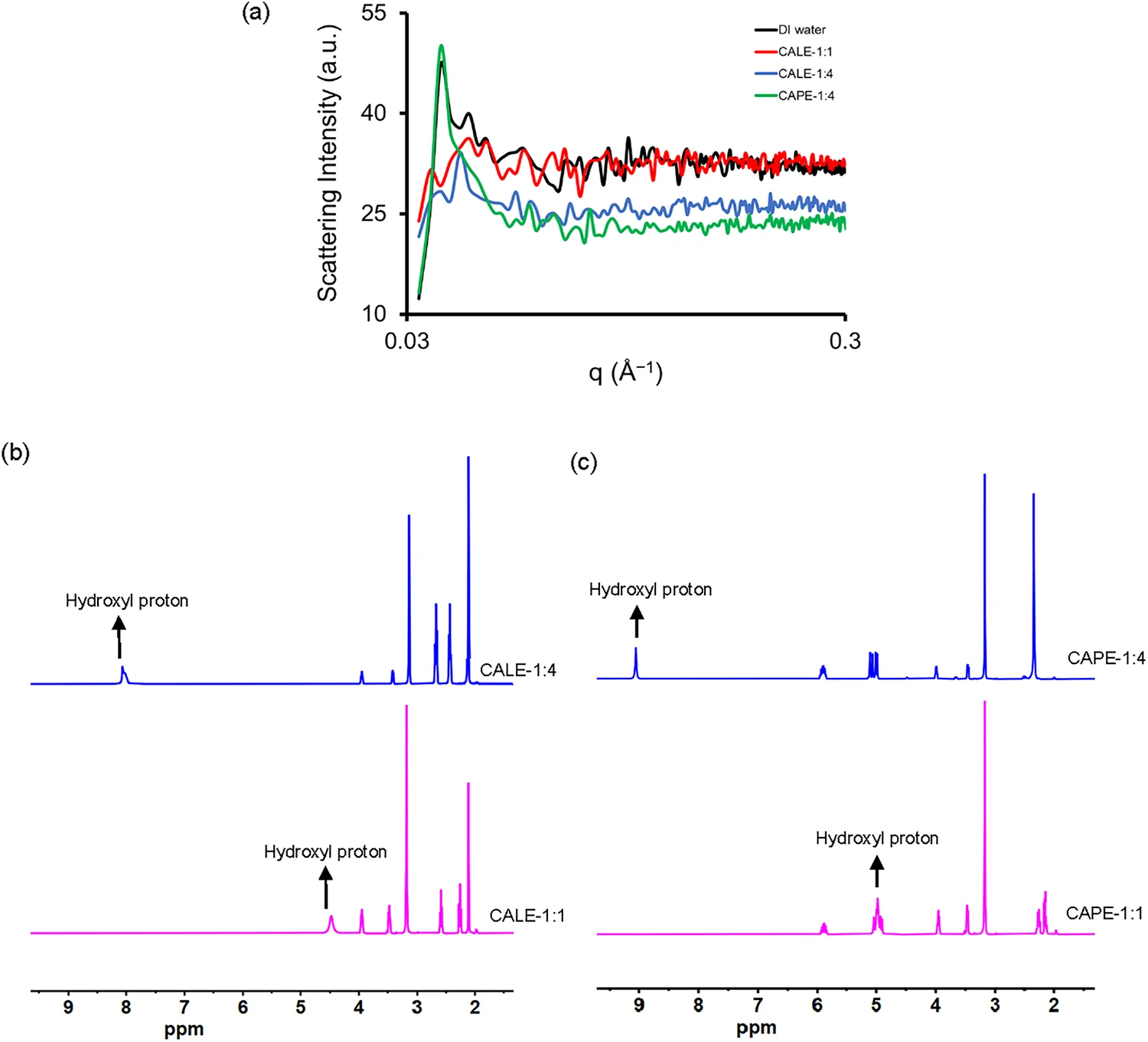
(a) Small-angle X-ray scattering (SAXS) profiles of 400 mM aqueous solutions of CALE-1:1, CALE-1:4, and CAPE-1:4, compared with deionized (DI) water. Scattering intensities were corrected using the measured transmitted-beam intensity relative to the empty-beam intensity and are shown as transmission-corrected scattering intensities. No pronounced low-(q) scattering enhancement or aggregation feature was observed for either ionic liquid relative to water, indicating the absence of SAXS-detectable nanoscale aggregates under the experimental conditions. (b) ^1^H NMR spectra of CALE-1:1 and CALE-1:4 acquired in DMSO-*d*_6_, highlighting the choline hydroxyl proton resonance. (c) ^1^H NMR spectra of CAPE-1:1 and CAPE-1:4 acquired in DMSO-*d*_6_, highlighting the choline hydroxyl proton resonance. DMSO-*d*_6_ was employed to minimize proton exchange and ensure observation of the hydroxyl resonance. In both systems, increasing the carboxylic acid content from the 1:1 to the 1:4 composition results in a downfield shift of the choline hydroxyl proton, consistent with changes in the local hydrogen-bonding environment. Together, the SAXS and NMR data indicate that the enhanced fluorescence observed for the 1:4 compositions is not accompanied by detectable nanoscale aggregation and correlates with changes in the local hydrogen-bonding environment.

Although SAXS revealed no evidence of nanoscale aggregation, the viscosity measurements indicated that fluorescence enhancement accompanies changes in the local intermolecular environment. In both IL series, increasing the carboxylic acid content simultaneously increased the viscosity and fluorescence intensity. Because viscosity in hydrogen-bond-rich ILs reflects the strength and extent of intermolecular interactions,^43,44^ ^1^H NMR spectroscopy was employed to probe the molecular environments associated with the fluorescence enhancement. Spectra were acquired in DMSO-*d*_6_ to reduce exchange broadening and permit the direct observation of the choline hydroxyl resonance. Because the ^1^H NMR and fluorescence measurements were performed in different solvent environments, the hydrogen bonding changes observed by ^1^H NMR may not directly reflect those present in the aqueous fluorescence samples. Nevertheless, in both IL series, increasing the acid content produced a pronounced downfield shift of the hydroxyl proton signal, from approximately 4.4 to 7.9 ppm in CALE and from approximately 5.0 to 8.1 ppm in CAPE (Figure 3b,c). These substantial deshielding effects indicate composition-dependent changes in the local environment of the choline hydroxyl proton and are consistent with changes in hydrogen-bonding interactions under the ^1^H NMR conditions. Within each IL series, increasing the carboxylic acid content shifts the hydroxyl chemical shift and increases the viscosity, accompanied by an increase in fluorescence intensity. However, the relationships among these observables differ between the CALE and CAPE. For example, CAPE-1:1 exhibits a slightly higher viscosity and a more downfield-shifted hydroxyl resonance than CALE-1:1, yet it displays substantially weaker fluorescence. Thus, neither viscosity nor hydroxyl deshielding consistently tracks the fluorescence behavior across the two IL series. Collectively, the SAXS, viscosity, and ^1^H NMR measurements show that the observed fluorescence does not require SAXS-detectable nanoscale aggregation and that fluorescence changes accompany composition-dependent changes in the local intermolecular environment. The different responses of CALE and CAPE further demonstrate that the carboxylic acid structure and composition distinguish the photophysical behavior of the ILs examined in this study.

### 3.2 Structural and Solvent Variations Influence Excitation-Dependent Photophysical Behavior

Excitation-dependent emission is a widely recognized characteristic of many nonconventional fluorophores, where multiple emissive states, heterogeneous local environments, or distinct through-space electronic interactions can be selectively populated under different excitation conditions.^8,34^ We therefore examined whether the ILs exhibit similar excitation-dependent photophysical behavior. Owing to their enhanced fluorescence relative to the corresponding 1:1 compositions, CALE-1:4 and CAPE-1:4 were selected for detailed photophysical investigations. CALE-1:4 exhibits pronounced excitation- and solvent-dependent emission behavior (Figure 4). In water, methanol, acetonitrile, and dimethyl sulfoxide (DMSO), excitation at 320 nm yields the most intense fluorescence, with a dominant emission band centered near 420–430 nm. Increasing the excitation wavelength broadens the emission profile, shifts emission toward longer wavelengths, and decreases the overall fluorescence intensity. This effect is most pronounced in DMSO (Figure 4d), where a distinct long-wavelength emission band centered near 500–530 nm emerges alongside the 420–430 nm band. Under 320 nm excitation, the higher-energy emission dominates, whereas excitation between 340 and 400 nm progressively enhances the lower-energy band. The emergence of this well-resolved dual-emission behavior in DMSO indicates that the solvent environment strongly influences the relative populations and relaxation pathways of the emissive states. CAPE-1:4 exhibits an even more pronounced excitation-dependent response and a markedly different emission profile from that of CALE-1:4 (Figures 4 and 5). Whereas CALE-1:4 is dominated by emission centered near 420–430 nm (Figure 4), CAPE-1:4 displays two well-separated emissive regions: a higher-energy band near 330–350 nm and a lower-energy band spanning approximately 500–530 nm (Figure 5). The relative intensities of these bands depend strongly on excitation wavelength. Excitation at 280–300 nm predominantly populates the short-wavelength emissive state, whereas excitation at longer wavelengths progressively enhances the lower-energy emission. This behavior is particularly evident in water and methanol (Figure 5a,b), where the two emissive regions can be selectively accessed simply by varying the excitation wavelength. Such excitation-dependent dual emission is consistent with the presence of multiple emissive states or competing excited-state relaxation pathways that differ in energy and relaxation dynamics.^45–48^ Particularly striking is the contrast between the excitation-dependent responses of CALE-1:4 and CAPE-1:4, given that the two ILs share a common choline cation and differ only in the functional group identity of their carboxylic acid component. When considered alongside the viscosity and ^1^H NMR results, these observations suggest that subtle structural variations alter the local intermolecular environment and, consequently, reshape the excited-state landscape accessible to the system. The ability of such a modest structural modification to produce substantial changes in emission wavelength, excitation dependence, and relative emissive-state populations underscores the sensitivity of nonconventional fluorescence to molecular-level organization. Similar excitation-dependent dual-emission behavior has been reported in a variety of nonconventional fluorophores, where multiple emissive channels arise from distinct clustered states, heterogeneous local environments, or different through-space interaction pathways.^46,48,49^

**Figure 4:**
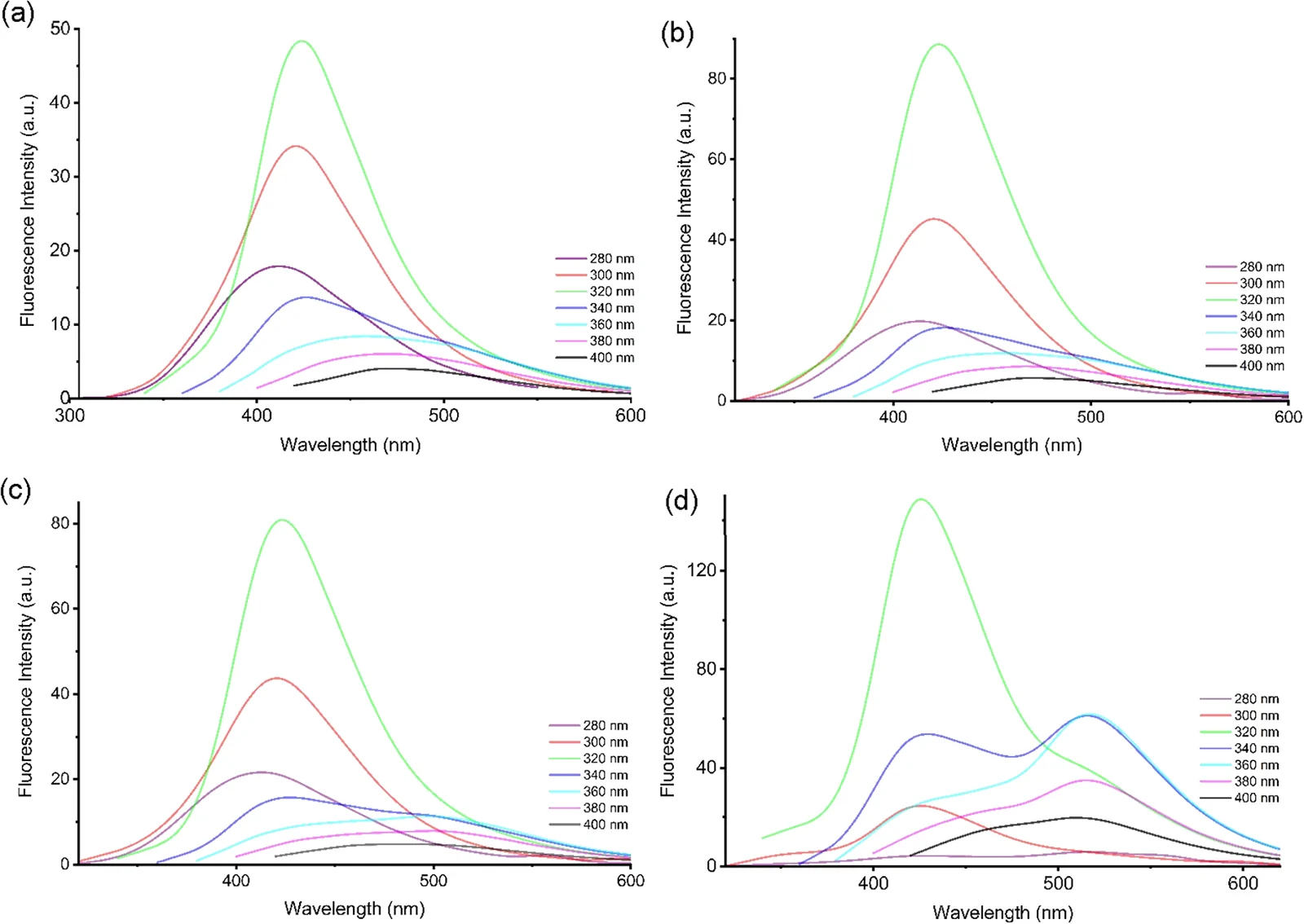
Excitation-wavelength-dependent fluorescence emission spectra of 400 mM solutions of CALE-1:4 (1 mol choline:1 mol levulinate:3 mol levulinic acid) in (a) water, (b) methanol, (c) acetonitrile, and (d) dimethyl sulfoxide (DMSO). The emission profiles exhibit pronounced solvent- and excitation-wavelength-dependent behavior. In water, methanol, and acetonitrile, CALE-1:4 displays a dominant emission band centered near 420–430 nm with maximum fluorescence intensity observed upon excitation at 320 nm. In DMSO, the emission spectrum undergoes a marked bathochromic shift and broadening, with the emergence of a long-wavelength emission band centered near 520 nm that becomes increasingly prominent at longer excitation wavelengths, indicating the presence of multiple emissive states or solvent-dependent excited-state relaxation pathways. Excitation wavelengths (280–400 nm) are color-coded as indicated in the legends. Fluorescence emission spectra were acquired on a Cary Eclipse fluorescence spectrophotometer using a 2 mm quartz cuvette with excitation and emission slit widths of 5 nm, a scan rate of 600 nm min^–1^, an averaging time of 0.1 s, and a data interval of 1 nm. All measurements were performed at a CALE-1:4 concentration of 400 mM.

**Figure 5:**
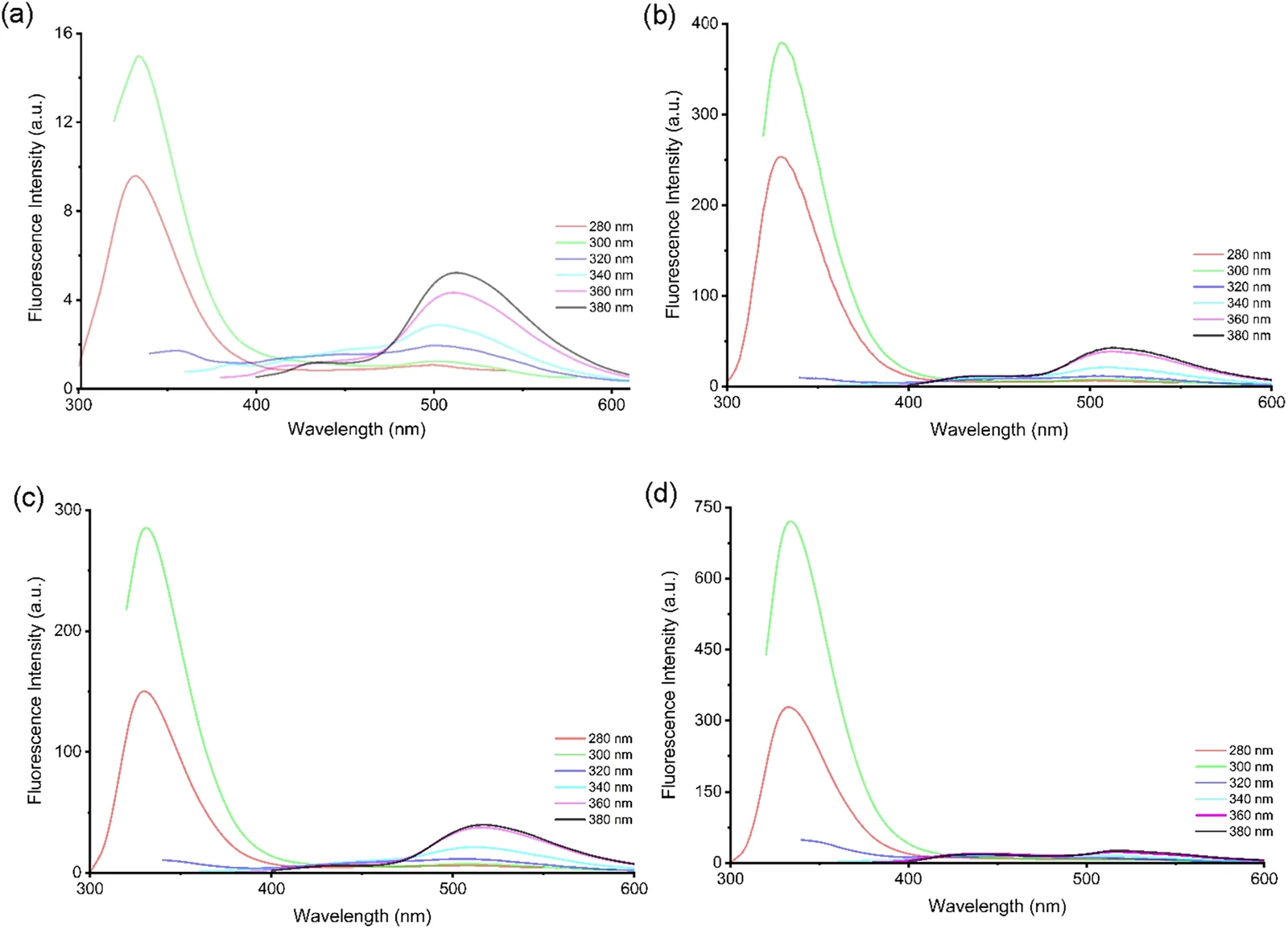
Excitation-wavelength-dependent fluorescence emission spectra of 400 mM solutions of CAPE-1:4 (1 mol choline:1 mol 4-pentenoate:3 mol 4-pentenoic acid) in (a) water, (b) methanol, (c) acetonitrile, and (d) dimethyl sulfoxide (DMSO). CAPE-1:4 exhibits pronounced solvent- and excitation-wavelength-dependent fluorescence characterized by two distinct emission regions centered near 330–340 nm and 500–530 nm. Excitation at 300 nm produces intense short-wavelength emission, whereas increasing the excitation wavelength progressively enhances the long-wavelength band, particularly in water, indicating the presence of multiple emissive states or excitation-selective relaxation pathways. The relative intensity of the long-wavelength emission decreases with decreasing solvent polarity, suggesting that the lower-energy emissive state is stabilized by specific solute–solvent interactions and/or heterogeneous local environments within the ionic liquid network. Excitation wavelengths (280–380 nm) are color-coded as indicated in the legends. Fluorescence emission spectra were acquired on a Cary Eclipse fluorescence spectrophotometer using a 2 mm quartz cuvette with excitation and emission slit widths of 5 nm, a scan rate of 600 nm min^–1^, an averaging time of 0.1 s, and a data interval of 1 nm. All measurements were performed at a CAPE-1:4 concentration of 400 mM.

A potential concern when interpreting excitation-dependent emission is that it may arise from concentration-dependent optical effects, such as inner-filter effects, reabsorption, and scattering, rather than from intrinsic photophysical processes. Because inner-filter and reabsorption effects become more pronounced in highly absorbing samples, dilution and reduced optical path lengths are commonly used to minimize these effects and assess their contribution to the observed fluorescence behavior.^50^ This consideration is particularly relevant for the 400 mM IL solutions, which exhibit appreciable absorbance at the excitation wavelength, especially the CALE systems (Figures SI1, and SI2). Accordingly, a 2 mm path-length cuvette was used for all fluorescence measurements to reduce inner-filter and reabsorption effects. To assess the extent to which inner-filter and reabsorption effects contribute to the observed fluorescence behavior, CALE-1:4 emission spectra were collected at substantially lower concentrations (40 and 4 mM) using a 2 mm path-length cuvette (Figure 6a,b). A corresponding dilution analysis of CAPE-1:4 was limited by its weaker fluorescence intensity (Figure 5a), as further dilution reduced the emission to levels that could not be reliably distinguished from the background. For CALE-1:4, excitation-dependent fluorescence remained evident at both concentrations, despite the substantial reduction in optical density (Figure S2). Although the shifts in emission maximum were not strictly monotonic, both the spectral profile and the wavelength of maximum emission intensity remained dependent on the excitation wavelength. Excitation-dependent fluorescence therefore persisted upon dilution, even as the overall emission intensity decreased markedly, particularly at 4 mM (Figure 6b). This pronounced loss in intensity is consistent with a common characteristic of many nonconventional fluorophores, which exhibit concentration-dependent emission.^13^ Although contributions from inner-filter effects and reabsorption to the absolute fluorescence intensity cannot be excluded, their reduction through dilution and a short optical path did not eliminate the excitation-dependent response. These observations, therefore, support excitation-dependent emission as an intrinsic photophysical feature of the IL system rather than a concentration-dependent optical artifact.

**Figure 6:**
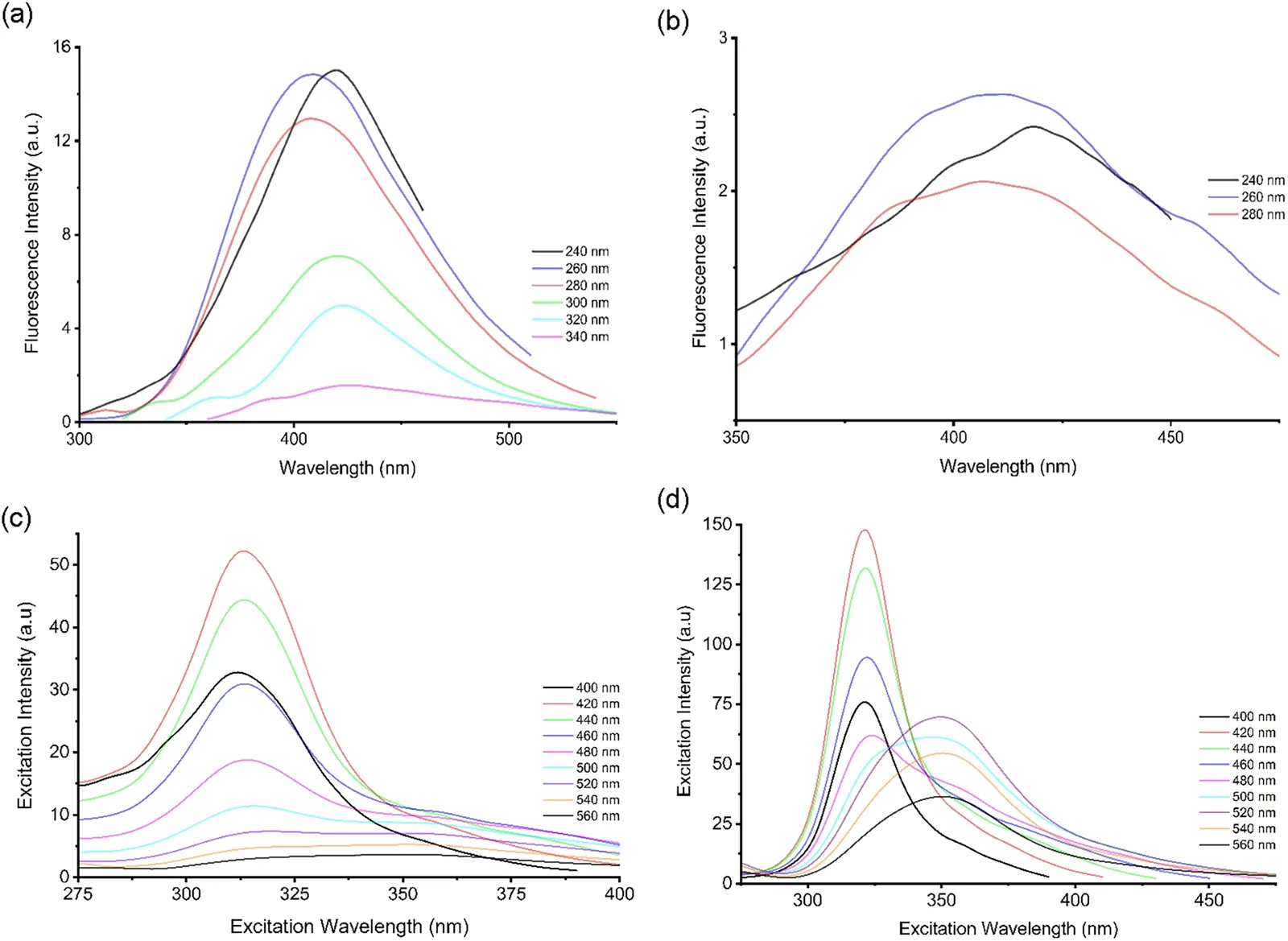
Excitation- and emission-dependent fluorescence behavior of CALE-1:4. (a,b) Emission spectra recorded at varying excitation wavelengths for CALE-1:4 at concentrations of 40 mM (a) and 4 mM (b). Excitation-dependent emission persists upon dilution and was measured using a 2 mm path-length cuvette, minimizing potential contributions from inner-filter effects and concentration-dependent optical artifacts. (c,d) Excitation spectra collected at different monitored emission wavelengths for CALE-1:4 in water (c) and DMSO (d). In water, the excitation spectra are dominated by a band centered near 310–320 nm with a weak shoulder extending toward 340–360 nm. In DMSO, this longer-wavelength excitation feature becomes substantially more pronounced and coincides with the emergence of more clearly resolved dual-emission behavior. Together, the excitation and emission data indicate that the wavelength-dependent fluorescence response is more consistent with the presence of multiple emissive populations or excited-state relaxation pathways than with a single structurally uniform emissive state.

To further probe the origin of the excitation-dependent fluorescence, excitation spectra were recorded at different monitored emission wavelengths for 400 mM CALE-1:4 (Figure 6c,d). In water, excitation spectra monitored between 400 and 460 nm are dominated by a broad excitation band centered near 310–320 nm (Figure 6c). However, when the monitored emission wavelength is shifted to 480 nm and above, a secondary long- wavelength excitation band emerges as a shoulder extending into the 350–380 nm region. Notably, the appearance of this band coincides with the emergence of the long-wavelength emission band in the excitation-dependent fluorescence spectra (Figures 6c and 4a). The effect becomes even more pronounced in DMSO (Figure 6d). Whereas shorter-wavelength emission is predominantly excited in the 310–320 nm band, monitoring emission between 480 and 560 nm reveals a well-resolved secondary excitation band at longer wavelengths. The emergence of this additional excitation feature parallels the distinct dual-emission behavior observed in DMSO (Figure 4d), where emission bands centered near 430 and 520 nm are clearly resolved. These observations are consistent with the characteristic excitation-dependent behavior of nonconventional fluorophores.^8,34^ They indicate that different regions of the emission spectrum arise from distinct excitation features, supporting the presence of multiple emissive populations or competing excited-state relaxation pathways rather than a single homogeneous emissive state.

Ultrafast transient absorption measurements provide further insight into the excited-state processes underlying the fluorescence of the ILs. Following excitation at 320 nm, 400 mM aqueous solutions of both CALE-1:4 and CAPE-1:4 exhibit pronounced spectral evolution within the first picosecond, characterized by the rapid development and decay of transient features across the probe window (Figure 7a,b). The transient spectra vary continuously with pump–probe delay time, whereas the kinetic traces extracted at selected probe wavelengths exhibit distinct temporal responses rather than synchronous growth and decay (Figure 7c,d). These wavelength-dependent dynamics indicate rapid excited-state relaxation and spectral reorganization on the sub-picosecond time scale. Although the transient absorption data do not resolve the individual excited-state populations, the observed spectral evolution is consistent with a complex excited-state landscape rather than a single relaxation process. This behavior aligns with the excitation-dependent steady-state fluorescence and excitation spectra discussed above, as well as with descriptions of nonconventional fluorescence and clusteroluminescence involving multiple excited-state configurations or relaxation pathways.^7^ Future global or target analysis will be required to resolve the number and identity of the contributing populations and obtain species-associated spectra.

**Figure 7:**
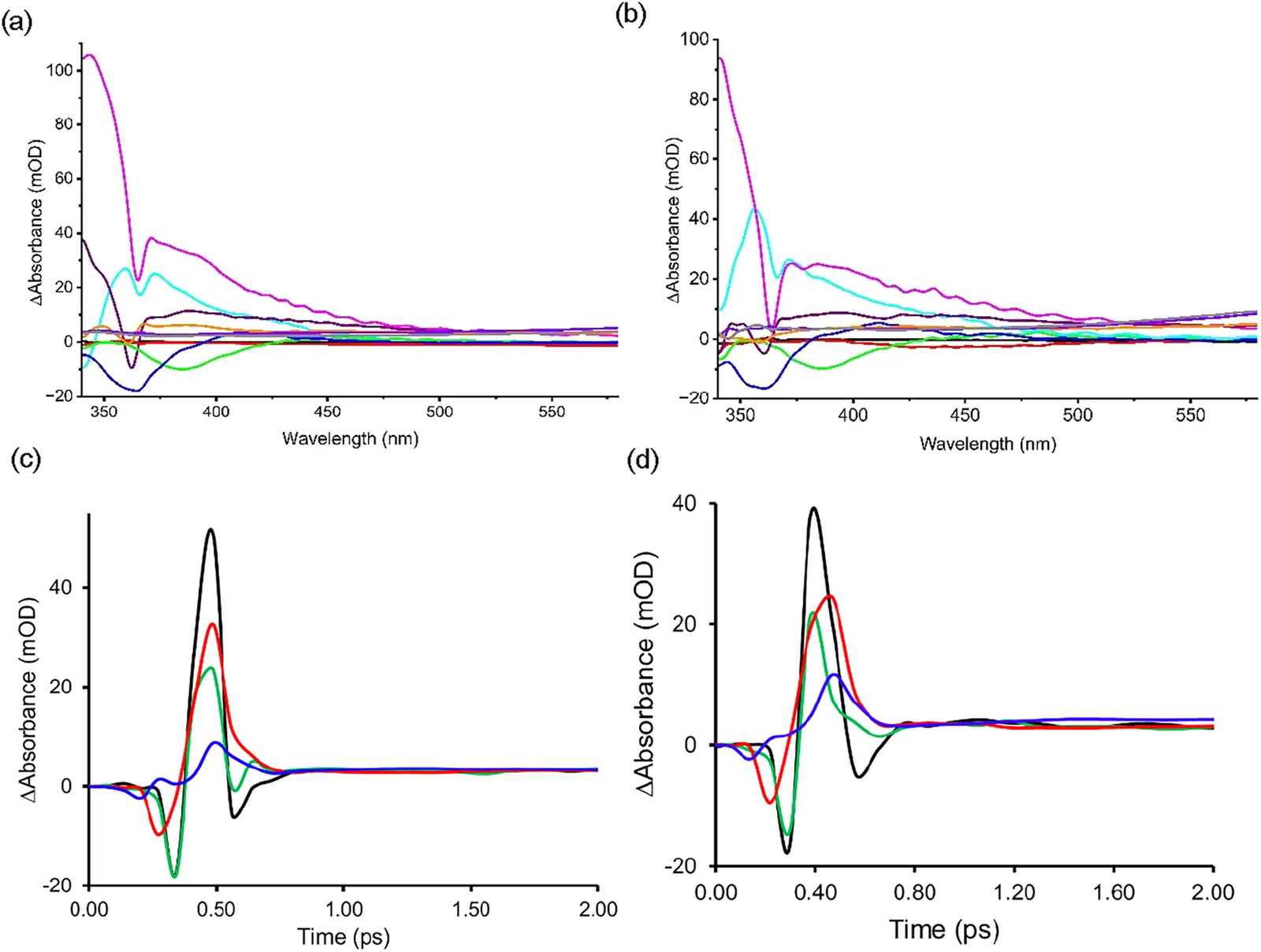
Ultrafast transient absorption spectroscopy of CALE-1:4 and CAPE-1:4 aqueous solutions following excitation at 320 nm. Top panels: transient absorption spectra of CALE-1:4 (a) and CAPE-1:4 (b) collected at selected pump–probe delay times, showing rapid spectral evolution within the first picosecond after excitation. Both systems exhibit time-dependent changes in the positions and amplitudes of transient features across the probe window, indicative of excited-state relaxation and spectral reorganization. Bottom panels: representative kinetic traces extracted at selected probe wavelengths for CALE-1:4 (c) and CAPE-1:4 (d). The wavelength-dependent and nonsynchronous temporal responses demonstrate that the photoexcited populations undergo rapid evolution on the sub-picosecond time scale. Together, the transient spectra and kinetic traces reveal a dynamic and heterogeneous excited-state landscape, consistent with the excitation-dependent fluorescence behavior observed in the steady-state measurements.

Time-resolved fluorescence measurements further demonstrate that the emissive states persist into the nanosecond regime (Figure 8). The fluorescence decays of 400 mM aqueous solutions of both CALE-1:4 and CAPE-1:4 are well described by biexponential reconvolution fits. CALE-1:4 exhibits lifetime components of 3.22 ± 0.01 and 14.32 ± 0.44 ns, corresponding to an amplitude-weighted average lifetime of 3.51 ns, whereas CAPE-1:4 exhibits lifetime components of 4.09 ± 0.06 and 12.13 ± 0.97 ns with an average lifetime of 4.48 ns. The biexponential decays provide further evidence of complex excited-state relaxation and are consistent with the excitation-dependent fluorescence and ultrafast transient absorption measurements. Notably, despite their close structural similarity, replacing levulinic acid with 4-pentenoic acid measurably alters the fluorescence decay kinetics, further demonstrating the sensitivity of excited-state relaxation to variations in carboxylic acid structure.

**Figure 8:**
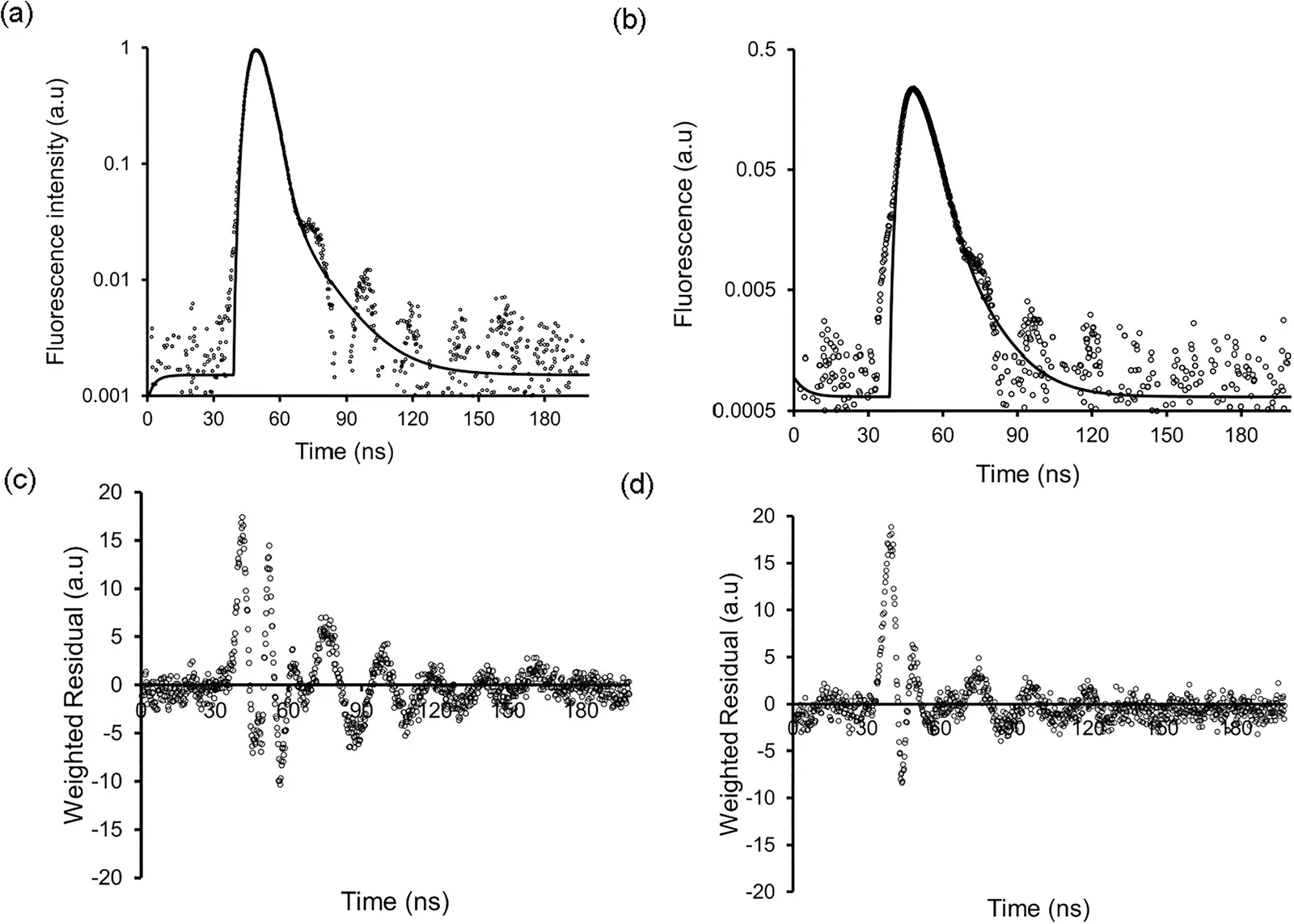
Time-resolved photoluminescence analysis of CALE and CAPE. (a, b) Semilogarithmic decay profiles recorded at the respective emission maxima following excitation at 320 nm, where symbols represent the experimental data and solid lines correspond to the biexponential reconvolution fits. (c, d) Weighted residuals of the corresponding fits, showing random distribution about zero with no systematic deviation, confirming the adequacy of the biexponential model and the reliability of the extracted lifetimes. Panels (a, c) correspond to CALE, while panels (b, d) correspond to CAPE.

Quantum-yield measurements further show that both ILs are weakly emissive in water, with values of 1.50 ± 0.06% for CALE-1:4 and 1.17 ± 0.02% for CAPE-1:4. These values fall within the range commonly reported for many unoptimized nonconventional fluorophores, whose emission typically arises from weak through-space electronic interactions and locally organized emissive states that remain susceptible to nonradiative relaxation.^12^ Rather than maximizing fluorescence efficiency, the objective of this study was to identify the molecular-level factors governing emissive-state formation and excited-state dynamics in these IL series. In this context, the observation that CAPE-1:4 exhibits a longer average fluorescence lifetime yet a lower quantum yield than CALE-1:4 is particularly informative, indicating that prolonged excited-state persistence does not necessarily translate into higher fluorescence efficiency. Instead, subtle changes in the functional groups embedded within the carboxylic acid framework shift the balance between radiative and nonradiative decay pathways. Taken together, the ultrafast transient absorption, time-resolved fluorescence, and quantum-yield measurements demonstrate that modest structural variations reshape the excited-state landscape and influence the pathways through which photoexcited populations evolve, relax, and ultimately emit.

## 4. Conclusion

In summary, this study demonstrates that carboxylic acid structure, composition, and solvent environment substantially influence the nonconventional fluorescence of choline–carboxylic acid ILs. Although CALE and CAPE differ only in the identity of the carboxylic acid component, they exhibit markedly different fluorescence responses, excitation-dependent emission profiles, and excited-state dynamics. Increasing the carboxylic acid content enhances the observed fluorescence intensity in both IL series, but no single measured property fully explains this behavior. Differences in excitationlight absorption may contribute to the observed intensities, while increased viscosity accompanies fluorescence enhancement within each series. However, neither viscosity nor ^1^H NMR-observed hydroxyl deshielding consistently tracks fluorescence across CALE and CAPE, and SAXS reveals no detectable nanoscale aggregation associated with the enhancement. Together, these observations point to a photophysical response that depends on carboxylic acid structure and composition rather than on any single measured physicochemical parameter. Steady-state and time-resolved spectroscopy further reveal excitation-dependent emission, wavelength-dependent ultrafast dynamics, and biexponential fluorescence decays, consistent with a complex excited-state landscape involving multiple emissive configurations or relaxation pathways. Within the molecular series examined here, replacing the carbonyl-containing levulinic acid component with the alkenyl-containing 4-pentenoic acid component produces substantial changes in fluorescence behavior and excited-state dynamics. Collectively, these findings establish choline–carboxylic acid IL hybrid solvents as a useful model system for investigating structure–photophysics relationships in nonconventional fluorescence.

## Supplementary Material



## Notes

^1^H and ^13^C NMR spectroscopic data for the choline–carboxylic acid ILs; UV–visible absorption spectra of CALE and CAPE compositions; and concentration-dependent UV–visible absorption spectra of CALE-1:4 (PDF)
