# Supplementary material for: Structure and Solvent Effects on Nonconventional Fluorescence in Choline–Carboxylic Acid Ionic Liquids Hybrid Solvents
Source: J Phys Chem B. 2026 Sep 9;130(38):10001–14. doi: 10.1021/acs.jpcb.6c05016 (PMC13618036; doi:10.1021/acs.jpcb.6c05016)
Supplement: Supplementary file 1 [file jp6c05016_si_001.pdf]

Structure and Solvent Effects on Nonconventional Fluorescence in Choline–Carboxylic Acid Ionic Liquids Hybrid Solvents

Solomon Owiredu[1], Aleah Berkebile[1], Grace Carr[1], Tashinga Chigodora[1], Juan Martinez Soto[1], Nicole McGuire[1], Duran Lee,[1] Samuel A. Oluwole,[1] J.S. Raaj Vellore Winfred[2], Christian Agatemor[1*]

[1]Department of Chemistry, Bucknell University, Lewisburg, PA 17837, USA.

[2]Department of Chemistry and Biochemistry, Florida State University, Tallahassee, FL 32306, USA.

*Corresponding Author (ca024@bucknell.edu)

NMR Spectroscopic Data of ILs

CALE-1:1: ^{1}H NMR (DMSO-d_6, 400 MHz): δ 4.47 (OH), 3.94 (CH_2), 3.46 (CH_2), 3.16 (CH_3)$_3$, 2.58 (CH_2), 2.25 (CH_2), 2.10 (CH_3). ^{13}C NMR (DMSO-d_6, 100.6 MHz): δ 209.70 (CO), 176.00 (CO), 67.64 (CH$_2$), 55.41 (CH$_2$), 53.42 (CH$_3$)$_3$, 40.15 (CH$_2$), 32.29 (CH$_3$), 30.12 (CH$_2$) ppm.

CALE-1:4: ^{1}H NMR (DMSO-d_6, 400 MHz): δ 7.52 (OH), 3.83 (CH_2), 3.37 (CH_2), 3.08 (CH_3)$_3$, 2.56 (CH_2), 2.30 (CH_2), 2.02 (CH_3). ^{13}C NMR (DMSO-d_6, 100.6 MHz): δ 207.93 (CO), 175.20 (CO), 67.53 (CH$_2$), 55.65 (CH$_2$), 53.66 (CH$_3$)$_3$, 38.26 (CH$_2$), 29.68 (CH$_3$), 29.00 (CH$_2$) ppm.

CAPE-1:1: ^{1}H NMR (DMSO-d_6, 400 MHz): δ 5.88 (CH), 5.01 (CH), 4.97 (OH), 4.90 (CH), 3.94 (CH_2), 3.47 (CH_2), 3.17 (CH_3)$_3$, 2.27 (CH_2), 2.15 (CH_2). ^{13}C NMR (DMSO-d_6, 100.6 MHz): δ 175.97 (CO), 138.78 (CH), 114.53 (CH$_2$), 67.64 (CH$_2$), 55.63 (CH$_2$), 53.73 (CH$_3$)$_3$, 35.87 (CH$_2$), 29.97 (CH$_2$) ppm.

CAPE-1:4: ^{1}H NMR (DMSO-d_6, 400 MHz): δ 9.02 (OH), 5.88 (CH), 5.05 (CH), 4.96 (CH), 3.96 (CH_2), 3.43 (CH_2), 3.16 (CH_3)$_3$, 2.32 2(CH_2). ^{13}C NMR (DMSO-d_6, 100.6 MHz): δ 175.30 (CO), 138.14 (CH), 114.98 (CH$_2$), 67.39 (CH$_2$), 55.64 (CH$_2$), 53.61 (CH$_3$)$_3$, 34.63 (CH$_2$), 29.65 (CH$_2$) ppm.

CAVA-1:1: ^{1}H NMR (DMSO-d_6, 400 MHz): δ 5.00 (OH), 3.79 (CH_2), 3.42 (CH_2), 3.11 -(CH_3)$_3$, 1.88 (CH_2), 1.36 (CH_2), 1.17 (CH_2), 0.77 (CH_3). ^{13}C NMR (DMSO-d_6, 100.6 MHz): δ 177.87 (CO), 67.46 (CH$_2$), 55.43 (CH$_2$), 53.29 (CH$_3$)$_3$, 38.07 (CH$_2$), 28.92 (CH$_2$), 22.60 (CH$_2$), 14.13 (CH$_3$) ppm.

CAVA-1:4: ^{1}H NMR (DMSO-d_6, 400 MHz): δ 7.52 (OH), 3.82 (CH_2), 3.41 (CH_2), 3.10 -(CH_3)$_3$, 2.08 (CH_2), 1.41 (CH_2), 1.21 (CH_2), 0.80 (CH_3). ^{13}C NMR (DMSO-d_6, 100.6 MHz): δ 176.30 (CO), 67.53 (CH$_2$), 55.52 (CH$_2$), 53.54 (CH$_3$)$_3$, 35.09 (CH$_2$), 27.48 (CH$_2$), 22.26 (CH$_2$), 13.67 (CH$_3$) ppm.

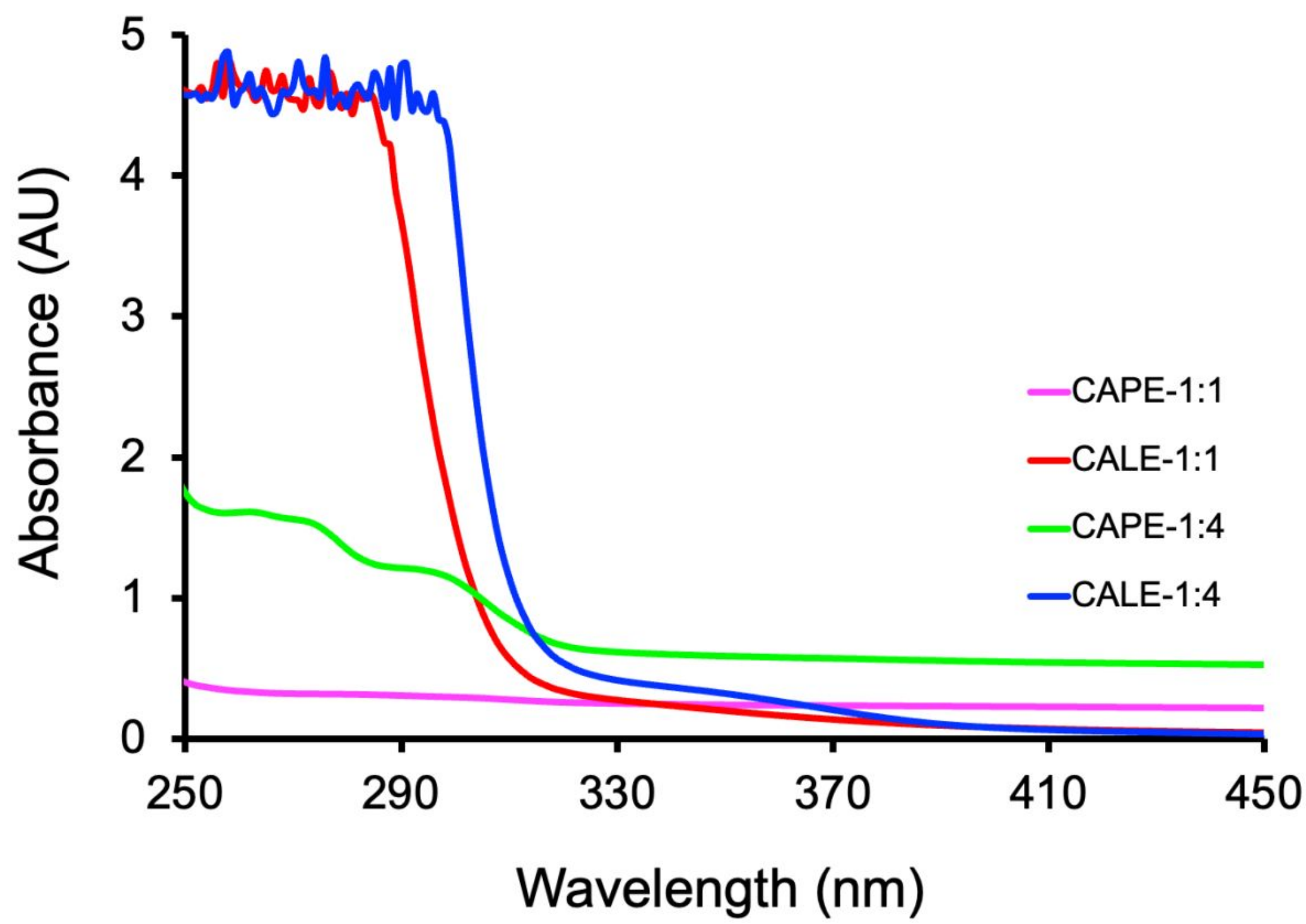

Figure S1. UV–visible absorption spectra of 400 mM aqueous solutions of CALE-1:1, CALE-1:4, CAPE-1:1, and CAPE-1:4, showing composition- and carboxylic acid structure-dependent differences in absorption across the measured wavelength range.

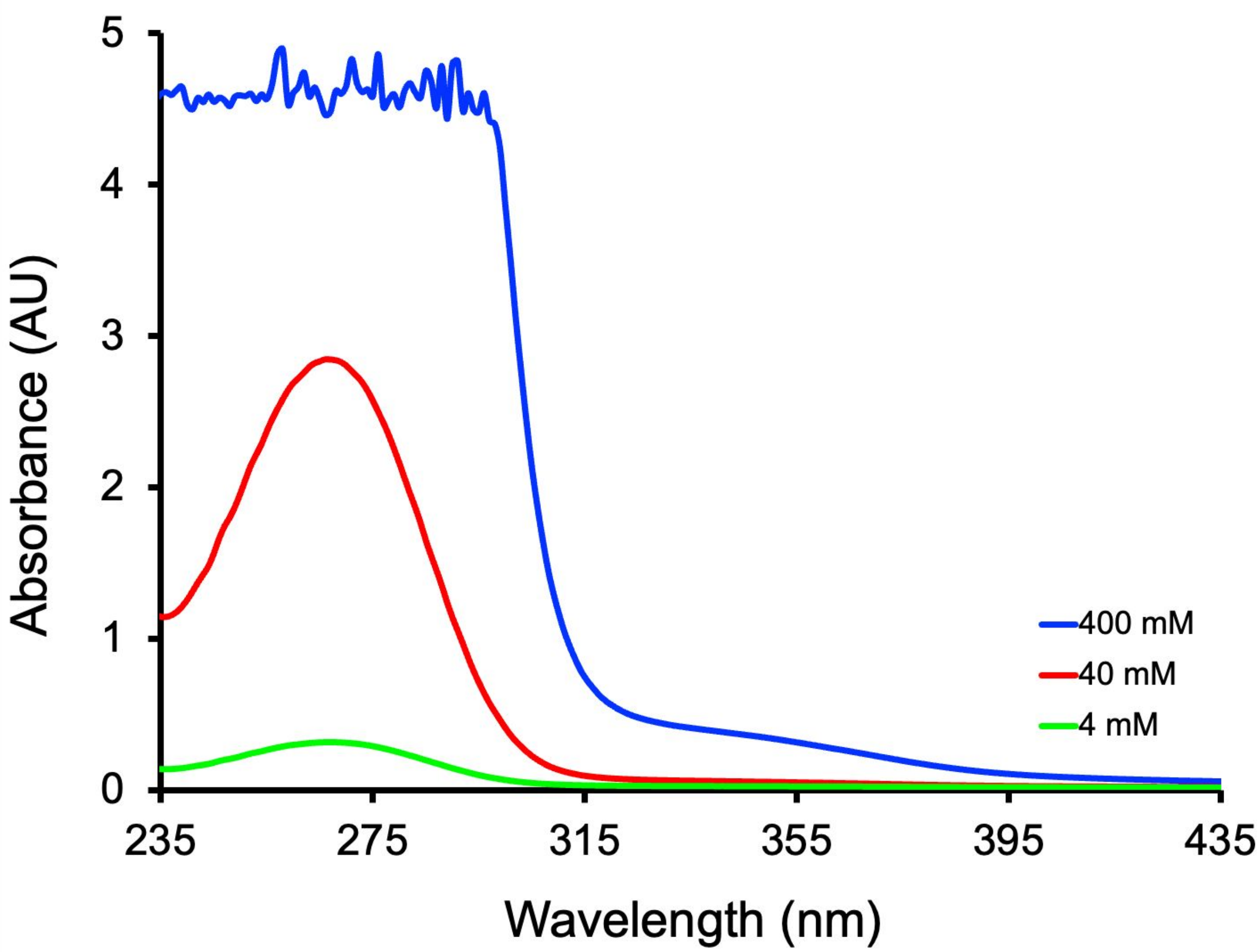

Figure S2. UV–visible absorption spectra of CALE-1:4 aqueous solutions at 400, 40, and 4 mM. Dilution substantially reduces the optical density across the measured wavelength range, providing the corresponding absorption conditions for the concentration-dependent fluorescence measurements.
